# The Impact of Continuing Bonds Between Pet Owners and Their Pets Following the Death of Their Pet: A Systematic Narrative Synthesis

**DOI:** 10.1177/00302228221125955

**Published:** 2022-09-07

**Authors:** Ben Hughes, Beth Lewis Harkin

**Affiliations:** 1 Faculty of Health and Wellbeing, 1796 University of Bolton, Bolton, UK; 2 School of Nursing, Faculty of Health and Wellbeing, 1796 University of Bolton, Bolton, UK

**Keywords:** pet loss, grief, continuing bonds, attachment, disenfranchised grief

## Abstract

When a pet dies, owners can experience similar levels of grief as when a human dies. Previous research indicates the role of continuing bonds (CB) when a pet is alive. To understand the impact of these bonds after the pet has died, we conducted a systematic narrative synthesis according to the Preferred Reporting Items for Systematic Reviews and Meta-Analyses guidelines (PRISMA). Findings were heterogenous, yet there were still parallels in the literature. CB can sometimes aggravate and intensify grief experiences, particularly when pet grief is perceived as disenfranchised grief. However, identifying appropriate bonds can be useful to moderate the intensity of grief and be a valuable mechanism of support. CB can also help post-traumatic growth of owners.

## Background

Over half the world’s population own a pet (Growth for Knowledge, 2016b, 2016a) and pet ownership is increasing (Sabin, 2018). Pets are often considered an important member of the family (Baydak, 2000; Dale, 2016; Dodgson, 2019; Hess-Holden et al., 2017; Podrazik et al., 2000; Stokes et al., 2002) and both their loyalty and the emotional and social support they provide is an integral part of a healthier lifestyle (Allen et al., 2000; Bradshaw, 2017; Compitus, 2019; Green et al., 2018; PetSecure, 2020; Podrazik et al., 2000; Waltham Foundation, 2020). This relationship between owners and pets may be particularly apparent following the Covid-19 pandemic. The first lockdown in the United Kingdom (UK) in 2020 led to an increase in pet enquiries and ownership (Battersea, 2020; Fox, 2020; Pets4Home, 2020; Waltham Foundation, 2020). Although many pets were abandoned or handed back to charities for rehoming, a lot were kept as loving companions (Battersea, 2020; Pets4Home, 2020; Waltham Foundation, 2020).

Axelrod (2020) recognizes the death of a pet can be painful and cause a range of emotional responses, such as anxiety, stress, shame, ambiguous grief, complicated grief, and even traits of psychopathology and trauma (Axelrod, 2020; Compitus, 2019; Hess-Holden et al., 2017; Sable, 2013; Taniyama et al., 2019). These emotions can lead to loneliness, isolation, and even suicidal thoughts (Antonacopoulos et al., 2010; Baydak, 2000; Hess-Holden et al., 2017; Sharkin & Knox, 2003; Taniyama et al., 2019). Such responses are often attributed to a lack of societal understanding and result in disenfranchised grief (Baydak, 2000; Chur-Hansen, 2010; Compitus, 2019; Cordaro, 2012; DiNicolantonio et al., 2018; Hess-Holden et al., 2017; Lagoni et al., 1994; Margolies, 1999; Sharkin & Knox, 2003; Taniyama et al., 2019). The term “disenfranchised grief” was first coined by Doka to recognize that some grief “is not or cannot be openly acknowledged, publicly mourned, or socially supported” (Doka, 1989, p. 4). Research indicates responses to the death of a pet positively correlate with the death of a human (Archer & Winchester, 1994; Hewson, 2014; Stokes et al., 2002), thereby signifying the importance of understanding the impact of a pet dying.

### Attachment Theory, Grief, and Continuing Bonds

The broad framework of Bowlby’s (1969, 1980) attachment theory is useful to understand grief responses of humans when their pet has died. According to Bowlby, humans have an innate need for attachment and close proximity to other people for safety, security, comfort, and support (Bowlby, 1969, 1980; Mikulincer & Shaver, 2022). The death of a loved one can therefore represent the struggle of detaching from them and the pain bereaved people experience and feel is what we term “grief.” Consequently, this theory helps understand functions, meaning, and disorders of attachment relationships and the attempt to maintain bonds with the deceased (Margolies, 1999; Sharkin & Knox, 2003). Continuing bonds (CB) are an effort to maintain this emotional attachment, or connection, following death and therefore represent a continuation of that attachment and an attempt to manage grief (Habarth et al., 2017; Packman et al., 2011). Exploring the role of CB as an ongoing relationship with the deceased helps understand this connection as either adaptive or maladaptive in the grieving process (Sirrine et al., 2018).

Previous research has identified that attachment to pets can provide comfort and reduce feelings of isolation and loneliness during stressful life events such as divorce and when owners are unwell or isolated (Allen et al., 2000; Sable, 1995, 2013). This bond also has a positive impact on physical and psychological wellbeing by creating a significant “other” to share life with (Sable, 2013). The unconditional relationship offered by pets (Archer, 1997) indicates the positive correlation between strength of attachment and the level of grief experienced after the pet dies (Antonacopoulos et al., 2010; Hunt & Padilla, 2006; Joseph et al., 2019; Noonan, 2008). However, there is a shortage of research exploring CB between pet owners and their dead pets and there is no consensus about the impact of this attachment after a pet has died (Antonacopoulos et al., 2010; Joseph et al., 2019). Similarly, there is a lack of research about support needs for bereaved owners. Therefore, the aim of this review is to identify major themes in the literature to explore the impact of CB between pet owners and their pets following the death of the pet and identify mechanisms of support for grieving pet owners. This synthesis has three key objectives:

1. To identify major themes in the literature to explore the role of CB between a pet owner and their pet.
2. To investigate what the literature reveals about the impact of CB between a pet owner and their pet.
3. To utilize the results of the narrative synthesis to understand the support needs of grieving pet owners.

This is the first-known study to synthesize the literature on this topic. Following the impact of the Covid lockdowns on pet ownership, it is a timely summary of existing research to benchmark current understanding of key areas.

## Method

The literature search comprised published resources and included the following databases: CINAHL Complete (Cumulative Index to Nursing and Allied Health Literature); MEDLINE (Medical Literature Analysis and Retrieval System Online); PubMed (Public MEDLINE); ProQuest Central, Oxford Reference Online; ProQuest Ebook Central; Royal College of Nursing Journals; Taylor and Francis Online; Sage Journals; JSTOR; Wiley Interscience; ScienceDirect; Emerald Journals; and Scopus. Searches will be run for additional literature on Google Scholar and OpenGrey. Searches were run in May 2020 and again in October 2020 to check for consistency. The review of the literature was conducted by both authors, with disagreements discussed and resolved between them. A third colleague was available to help resolve any disagreement about the literature but was not needed. There were not any restrictions on the date range of articles published to ensure all relevant literature was included in the study. The experiences of pet owners and research related to support needs was reviewed. The synthesis was focused on primary, peer-reviewed research rather than literature reviews or secondary sources to improve reliability of accurate findings being included.

An initial search of the literature indicated some key terms used in previous studies in the broad topic area. These terms were used for this study to ensure relevant and appropriate literature was included in the search and their synonyms were used to help identify broader relevant literature. When searching for literature, truncation was added using an asterisk at the end of words to encompass different spellings or word endings, such as “bereave*.” Where available, an advanced search strategy was implemented to allow words to be searched in relation to their proximity to one another. The search terms used in the literature review were: Pet **OR** *companion* **AND** Died **OR** continuing bonds **OR** attach* **AND** Grie* **OR** bereave* **OR** Mourn*. Studies were selected for review based on clear inclusion and exclusion criteria (see Table 1).

**Table 1.** Inclusion and Exclusion Criteria for Study Selection.

Inclusion criteria	Exclusion criteria
• Sources written in English • Studies focused on domesticated pets who have died • Studies focused on the continuing bonds between pet owners and their pets • Studies focused on the role of continuing bonds/attachment and the grief/bereavement experiences of pet owners	• Sources written in languages other than English • Sources exploring relationships between pet owners and their pets where the pet is still alive • Studies focused on animals which are not domesticated pets, such as wild animals, working animals, or agricultural animals • Studies focused on relationships other than continuing bonds between pet owners and their pets • Studies exploring experiences other than continuing bonds/attachment and grief/bereavement experiences

The search strategy was adopted from previous research which included heterogenous studies (Hughes et al., 2018) and adhered to the narrative synthesis guidelines recommended by Popay et al. (2006). This process ensured consistency, clarity and transparency within the data extraction and management process. Findings will be presented thematically in line with previous similar research (Adams et al., 1999; Baydak, 2000; Dunn et al., 2005; Lavorgna & Hutton, 2019; Packman et al., 2012; Rémillard et al., 2017).

**Figure 1.**
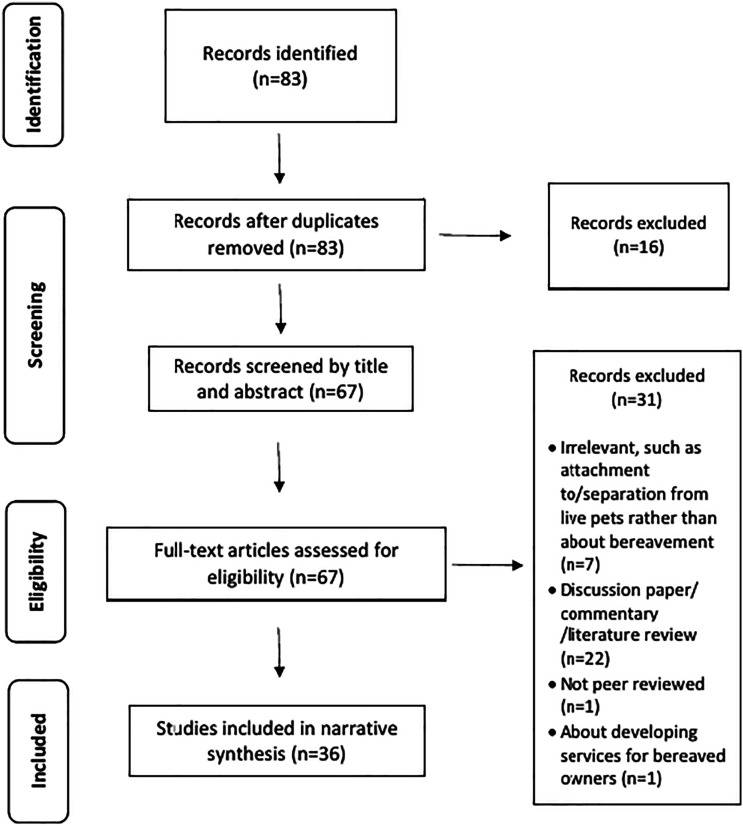
Summary of the literature search.

The synthesis was registered with PROSPERO (PROSPERO registration number: CRD42020177912). The Preferred Reporting Items for Systematic Reviews and Meta-Analysis (PRISMA) (Liberati et al., 2009; Stewart et al., 2015) is used to present the literature search (see Figure 1).

Hawker et al.’s (2002) literature assessment framework (Appendix 1) and literature scoring system (Appendix 2) were used to assess the quality of the studies included in the synthesis. The assessment framework allows literature to be scored (9 very poor; 36 very good) to indicate the methodological rigor of each study (Hawker et al., 2002). This scoring process gave a clear indication of the strengths and weaknesses of each study and so provided clarity, transparency, and rigor in the quality assessment process. No studies were rejected because of poor methodological quality and all studies were included in the review.

## Findings

### Overview of the Studies

A detailed summary of study findings can be found in Appendix 3. Publication dates of the studies ranged from 1994 to 2020 and the majority were broadly quantitative. The studies were from various countries across the world but were primarily from the United States. Studies differed in their reporting of demographic details of participants, with few or no demographic details were provided in a third of studies. A minority of studies concentrated on adolescents or young adults, including university students; or children, or children and their parents. When gender was reported, the majority of participants were female. When race/ethnicity of participants were identified, the majority of participants identified as White/Caucasian. A quarter of studies were focused solely on the death of pet dogs and/or cats, while other studies concentrated on the death of a pet dog or cat, with the addition of other animals such as birds, rabbits, guinea pigs, parrot, rat, reptiles, pony, horses, and ferrets.

Due to these reasons, the literature inevitably varied in quality. Factors which negatively impacted the quality of research included ineffective abstracts (Morris, 2012); a lack of clarity in the aim(s) of the study (Morris, 2012); poor description of data collection and/or sampling methods (Laing & Maylea, 2018; McCutcheon & Fleming, 2001; Morris, 2012; Redmalm, 2015; Rémillard et al., 2017; Rujoiu & Rujoiu, 2015); incomplete data analysis (Dunn et al., 2005; Morris, 2012); poor consideration of ethical issues and/or potential for bias (Archer & Winchester, 1994; Dunn et al., 2005; McCutcheon & Fleming, 2001; Morris, 2012; Redmalm, 2015; Rémillard et al., 2017; Rujoiu & Rujoiu, 2015; Wrobel & Dye, 2003); descriptive findings which lacked detail (Morris, 2012); or a lack of generalizability and/or discussion of implications for policy and practice (Archer & Winchester, 1994; McCutcheon & Fleming, 2001; Redmalm, 2015; Rémillard et al., 2017; Rujoiu & Rujoiu, 2015). Nevertheless, only a minority of papers (Archer & Winchester, 1994; Dunn et al., 2005; Laing & Maylea, 2018; McCutcheon & Fleming, 2001; Morris, 2012; Redmalm, 2015; Rémillard et al., 2017; Rujoiu & Rujoiu, 2015; Wrobel & Dye, 2003) were judged as poor in overall quality and none were considered very poor. All included papers added some valuable information to the synthesis.

Three main themes were identified in the literature from the global theme of continuing bonds: intensity of grief; support mechanisms and means of coping; and personal growth. The studies generally covered multiple themes and will be discussed below. A summary of the themes in the included literature is in Table 2:

**Table 2.** Summary of the Included Literature.

Paper	Theme 1 intensity of grief	Theme 2 support mechanisms and means of coping	Theme 3 personal growth
(Archer & Winchester, 1994)	X	X	
(Brown et al., 1996)	X	X	
(Brown & Symons, 2016)	X		
(Bussolari et al., 2018)	X	X	
(Dunn et al., 2005)		X	
(Eckerd et al., 2016)	X		
(Field et al., 2009)	X	X	
(Green et al., 2018)	X	X	
(Habarth et al., 2017)	X	X	X
(Hunt et al., 2008)	X		
(Hunt & Padilla, 2006)	X	X	
(Kaufman & Kaufman, 2006)		X	X
(King & Werner, 2011)	X	X	
(Krause-Parello & Gulick, 2013)	X	X	
(Laing & Maylea, 2018)	X	X	
(Lavorgna & Hutton, 2019)	X	X	
(Lee, 2016)	X	X	X
(Lee & Surething, 2013)	X	X	
(Luiz Adrian et al., 2009)	X	X	
(McCutcheon & Fleming, 2001)	X	X	
(Morris, 2012)		X	
(Packman et al., 2011)	X		X
(Packman et al., 2012)	X	X	
(Packman et al., 2014)	X	X	X
(Packman et al., 2017)		X	X
(Redmalm, 2015)	X		
(Rémillard et al., 2017)	X	X	X
(Rennard et al., 2019)	X	X	
(Rujoiu & Rujoiu, 2015)	X	X	
(Schmidt et al., 2020)	X	X	
(Testoni et al., 2017)	X	X	
(Tzivian et al., 2014)	X	X	
(Tzivian et al., 2015)	X	X	
(Wong et al., 2017)	X	X	X
(Wrobel & Dye, 2003)	X	X	
(Zilcha-Mano et al., 2011)	X		

### Theme 1––Intensity of Grief

The contribution of CB to the intensity of grief was a significant theme identified in the literature and was identified in 32 sources. This theme is divided into four relevant sub-themes below:

#### Grief as a destabilizing emotion

Nearly two thirds of respondents in one study described their animal as a “baby,” “child,” “best friend,” “companion,” or someone to be “loved” rather than just a “protector” or a “pet” (Archer & Winchester, 1994). Understandably, grief is a potentially destabilizing emotion which was exhibited in various behaviors ranging from numbness and/or disbelief to clinical depression, trauma, and post-traumatic stress disorder (Archer & Winchester, 1994; Hunt et al., 2008; Hunt & Padilla, 2006; King & Werner, 2011; Luiz Adrian et al., 2009; Packman et al., 2014; Redmalm, 2015; Wrobel & Dye, 2003; Zilcha-Mano et al., 2011). These feelings led to preoccupation with thoughts of the pet, poor concentration, avoidance behavior, loss of identity, self-reproach, and socialization (Archer & Winchester, 1994; Brown & Symons, 2016; Hunt et al., 2008; King & Werner, 2011; Krause-Parello & Gulick, 2013; Tzivian et al., 2015; Zilcha-Mano et al., 2011).

#### The impact of continuing bonds on grief

The evidence showed CB can intensify the grieving process by focusing on negative bonds, leading to severe grief (Field et al., 2009; Lavorgna & Hutton, 2019; Rémillard et al., 2017). In these situations, CB with the dead pet led to somatizing symptoms of grief similar to those for family members and friends (King & Werner, 2011; Tzivian et al., 2014). Consequently, the five stages of grief outlined by Kübler-Ross (1969) are present in the literature about pet death (Adams et al., 1999; Antonacopoulos et al., 2010; Baydak, 2000; Chur-Hansen, 2010; Compitus, 2019; Kolodny, 1991; Sharkin & Knox, 2003; Stokes et al., 2002).

Conversely, there is the potential for CB to mitigate the effects of grieving by decreasing feelings of loneliness. Rituals, memorials, memories, and dreams were identified as helpful coping mechanisms by continuing levels of attachment and reducing the intensity of grief (Krause-Parello & Gulick, 2013; Packman et al., 2012).

#### The character of grieving owners

White owners tended to treat their pet more like a family member than non-white owners and so the bond between them was felt more strongly when the pet died (Hunt & Padilla, 2006). However, the literature indicated owners aged 18–35 and over 60 are more likely to experience grief (McCutcheon & Fleming, 2001). Children and adolescents may also experience more intense grief, depending on their age, previous experiences of death, and level of attachment to the pet (Brown et al., 1996; Schmidt et al., 2020). Most literature indicated females form a stronger bond with their pet and so do experience grief more intensely than males (Brown & Symons, 2016; Eckerd et al., 2016; McCutcheon & Fleming, 2001; Testoni et al., 2017; Wrobel & Dye, 2003). However, the conclusions drawn in the literature were based on studies in which most participants were White, adult females and therefore may be the only conclusions to draw. Consequently, there is little understanding about the impact of CB on grieving non-White, non-female, younger pet owners, or bereaved people who have owned pets other than cats or dogs.

Other individual factors were identified as equally important when considering the role of CB and pet grief. For example, grief intensity was reportedly greater if the death was sudden and the person lived alone (Archer & Winchester, 1994). There was disagreement in the literature regarding the length of time attachment continued after the death of a pet. Periods for the peak of emotions ranged from 2 to 6 months (Krause-Parello & Gulick, 2013; McCutcheon & Fleming, 2001; Tzivian et al., 2014) and possibly up to a year after the pet’s death (Tzivian et al., 2014; Wrobel & Dye, 2003). Other research indicated grief peaked again approximately a year after the pet died, with up to 20% of pet owners reporting grief symptoms a year after the death (Hunt & Padilla, 2006; Wrobel & Dye, 2003). Therefore, recognizing the individual’s unique situation and their bond with the pet is felt important to understand the intensity of grief and how long it is experienced.

#### Disenfranchised grief

Grief experiences were further complicated because of disenfranchised grief. Although memorialization and rituals helped reduced the intensity of grief and stimulated personal growth, pet death was widely recognized as disenfranchised grief which had implications for both level of support and access to available support (Archer & Winchester, 1994; Lavorgna & Hutton, 2019; Packman et al., 2012; Rémillard et al., 2017; Tzivian et al., 2014, 2015). The understanding of pet bereavement was generally poorer and more trivialized in affluent societies, where grief was also linked to a hierarchy of animals (Laing & Maylea, 2018; Wong et al., 2017). This hierarchy has sometimes recognized the loss of dogs and cats as significant but the loss of other animals, such as fish and rats, has not received the same validation (Laing & Maylea, 2018). Some bereaved pet owners have described a “double disenfranchisement” whereby their feelings of grief for the pet have not been widely recognized by society and, in addition, their emotional connection to an animal considered lower down the hierarchy is considered abnormal (Laing & Maylea, 2018). Therefore, the literature indicates grieving owners should be supported by recognizing their unique needs and understanding that CB can intensify grief in some situations while alleviating it in other circumstances.

### Theme 2––Means of Coping and Support Mechanisms

As with Theme 1, the recognition of support and coping was a comprehensive theme identified in the literature and was covered by 33 of the eligible studies. Two sub-themes are outlined below:

#### Continuing bonds and religion as means of coping

Some owners maintained bonds with a dead pet through an existing or new pet (Redmalm, 2015; Wong et al., 2017). However, there was no conclusive evidence to indicate whether this reduced grief or simply delayed its onset. If the owner views their pet as a family member, replacement of one pet with another is also not as simple or as viable as it sounds. Focusing on “replanning” following the death of a pet was sometimes negative because of the permanent and inescapable nature of death (Green et al., 2018). Instead, owners who were oriented toward using CB as a method of self-compassion reported less intense grief, less frequent dismissive or negative social interactions, and better psychosocial functioning (Bussolari et al., 2018).

Evidence also suggested that grieving owners needed someone to talk to and a means to express their grief (Brown et al., 1996). Many owners sought to maintain CB by incorporating their dead pet into religious belief (Testoni et al., 2017). In this way, owners discovered positive expressions of religious coping to deal with their loss through seeking God’s love and care (Lee, 2016). Belief in an afterlife for people and animals was associated with stronger attachment and improved grief response because of a communal sharing of the relationship; conversely, belief in an afterlife for only people led to more intense grief and was considered a less effective coping mechanism (Testoni et al., 2017). Other owners believed their pet’s soul was in a worse place, such as hell, and so they engaged in negative forms of religious coping because they felt punished by God (Lee, 2016; Lee & Surething, 2013). Therefore, religion was sometimes associated with feelings of guilt or comfort, depending on whether the owner felt punished or sought God’s love (Lee, 2016; Lee & Surething, 2013; Testoni et al., 2017). Overall, evidence suggested owners who drew on both positive and negative aspects of religion coped with grief more effectively because of their search for meaning (Lee, 2016). For this reason, spirituality, or a more subjective process of searching for connectedness and contextualizing one’s own existence, was helpful to cope more effectively (Lee & Surething, 2013).

#### Social and professional support as a support mechanism

Social support was also identified as an important mechanism in which to share CB and maintain the owner’s quality of life following the death of their pet and validate emotions (Field et al., 2009; Packman et al., 2012, 2017; Tzivian et al., 2015). The greater the perceived levels of social support, the lower the reports of attachment anxiety and attachment avoidance, and vice versa (Field et al., 2009; King & Werner, 2011). Social support was particularly important for people living alone (Archer & Winchester, 1994; Hunt & Padilla, 2006); those with family or friends unable to offer support (Tzivian et al., 2015); and for children (Kaufman & Kaufman, 2006; Schmidt et al., 2020).

Other literature indicated that social networks were was less important in pet death than in human death (Field et al., 2009; Green et al., 2018), suggesting not all pet owners have the same level of attachment to their pet, feel the same intensity of grief, or benefit from the same support. Yet there were situations when grieving individuals were shown to have benefitted from professional support and treatment (Luiz Adrian et al., 2009).

Although many vets shared similar emotions to grieving owners (Rujoiu & Rujoiu, 2015), perceived reactions of vets to the existence of CB further negatively impacted grief (Rémillard et al., 2017). Vets were often the initial contact for owners and literature indicated they should be available to help owners self-manage their emotions, as well provide initial support and contacts, make phone calls the day after the death, and send a condolence letter (Morris, 2012; Rémillard et al., 2017; Tzivian et al., 2014, 2015). Social workers may provide additional support as a conduit between vets and owners (Dunn et al., 2005). However, evidence suggested a barrier to accessing support was created because healthcare professionals did not always accept the death of a pet family member in the same way they accepted the death of human family member (Wrobel & Dye, 2003). Consequently, professionals can sometimes reduce intensity of grief by recognizing CB and facilitating access to support. Conversely, they may amplify grief and create an obstacle to support by failing to understand the role of CB in pet bereavement.

### Theme 3––Personal Growth

Personal growth was identified as a relatively small but important theme in eight studies. Two sub-themes are outlined below:

#### Religion and spirituality

Appreciation for life was also reflected in psychological or spiritual growth. Bereavement highlighted the owner’s personal strength and so provided a meaning to life (Packman et al., 2017). Although the death was painful, facing difficult decisions around euthanasia or discovering coping mechanisms for the death helped some owners identify resilience they did not realize they had. Pet death encouraged other owners to channel their energies into being creative or productive and so helped refocus their attention. Coming to terms with death enabled the grieving to offer support and information to other people or volunteer to work with animals (Packman et al., 2014, 2017; Wong et al., 2017). Some owners also reported the death of a pet led to a strengthening of bonds with existing pets (Wong et al., 2017).

The multifaceted positive layers of religion and spirituality helped the search for meaning and validated feelings of purpose, although religious faith itself did not seem to be strengthened (Lee, 2016; Packman et al., 2017; Wong et al., 2017). Yet reflections on life and death provided meaning and inspiration to cherish what life still had to offer. For example, a strong bond with a pet helped bring some owners closer to other family members after the pet died (Packman et al., 2017; Wong et al., 2017).

#### Continuing bonds, attachment, and growth

Despite previous research indicating the negative impact of pet bereavement, evidence suggested CB might be beneficial for encouraging post-traumatic growth. CB helped the bereaved find value in the death of a pet and, in doing so, facilitated the search for comfort (Habarth et al., 2017; Packman et al., 2011; 2017). In this sense, CB acted as a regulating and adapting process (Packman et al., 2011).

A strong bond with a pet was a considerable factor in other aspects of personal growth. Using a range of positive CB associations was shown to have an impact on wider aspects of health, wellbeing, and relationships with the wider world (Habarth et al., 2017). Growth was reported in the expression of emotions such as empowerment, happiness, love, positive thinking, and coping behaviors (Rémillard et al., 2017; Wong et al., 2017). Higher levels of post-traumatic growth were also associated with lower levels of somatization and functional impairment (Habarth et al., 2017).

The bereaved also expressed meaning through verbal discussions and poetry (Kaufman & Kaufman, 2006), writing a eulogy (Rennard et al., 2019); and engaging in pet loss support groups and counseling (Dunn et al., 2005; Laing & Maylea, 2018; Lavorgna & Hutton, 2019; Rujoiu & Rujoiu, 2015; Tzivian et al., 2014; Wong et al., 2017). The strength of attachment to the pet reflected these expressions and fostered empathy and compassion for other people, as well as recognition and acceptance of the sympathy that was offered in the period after the pet had died (Packman et al., 2017). Validating expressions of CB also helped minimize symptoms of grief and their duration (Kaufman & Kaufman, 2006).

## Discussion

As far as it is known, this is the first systematic review to understand the impact of CB between pet owners and their pets following the death of the pet. Using a valid, reliable, and rigorous approach to searching for and analyzing the literature, three themes were identified.

In relation to the study’s first objective, the synthesis identified that the strength of the bond with a pet and the resulting impact of grief relates to pets being seen as family members. In relation to the study’s second objective, CB can make negative and positive impacts on the grieving experience. A strong bond with a pet can lead to a range of emotional responses and informal and formal social support can help maintain positive bonds and mitigate the effects of grief. The type and strength of impact is influenced by various factors, such as age, gender, previous grieving experiences, and the strength of the bond with the pet. At their extreme, owners can be debilitated and would benefit from professional support. Stereotypical ideations of owners and grief experiences are not particularly useful and have implications for the coping mechanisms available for grieving owners. Conclusions based on these findings reflect that most research has been conducted with participants who are predominantly White, female adults. Therefore, the current understanding about the impact of CB between a pet owner and a pet is based on a narrow population sample. The perspectives of different ethnicities and genders are not often included in previous research and so current literature is not representative of every pet owner. This is important because mechanisms of support depend on wider societal and professional attitudes. In relation to objective three of the study, informal support and some aspects of spirituality can identify and maintain positive aspects of CB. This basis can provide a foundation for finding value, creating meaning, strengthening relationships, or personal growth.

Overall, evidence suggests grieving owners would benefit from empathetic communication to validate and legitimize their feelings and so provide effective support (Rennard et al., 2019; Wong et al., 2017; Wrobel & Dye, 2003). This approach necessitates support from someone they trust to listen, understand, and validate their experiences, and help them develop coping mechanisms (Wong et al., 2017). The availability and quality of support provision depends in part on the attitudes of society and professionals. Opportunities to access appropriate external support are partially dependent on perceptions of disenfranchised grief. These perceptions are a barrier to seeking support as well as being a potential barrier to providing support.

Further research is needed to better understand the impact of CB on males, non-white populations, and different age groups following the death of a pet. Finally, amplifying discussions around pet grief will help to franchise grief experiences and create safe spaces to open conversations with those around us.

### Limitations and Strengths

There are several limitations to this review. A narrative synthesis aims to, and supports, the synthesis of heterogeneous studies, but the varied nature of the studies creates a potential for bias. Variation in terminology used to refer to pets was a challenge when searching for articles and discussing the impact of CB.

Despite the limitations above, the included studies and synthesis approach satisfactorily answer the review question. The design of the synthesis reduced the potential for bias and ensured the review was conducted rigorously and is replicable. Even with the heterogeneous nature of the studies, the findings appear similar and are applicable to a variety of owners and settings.

## Supplemental Material

Supplemental Material - The Impact of Continuing Bonds Between Pet Owners and Their Pets Following the Death of Their Pet: A Systematic Narrative Synthesis Supplemental Material for The Impact of Continuing Bonds Between Pet Owners and Their Pets Following the Death of Their Pet: A Systematic Narrative Synthesis by Ben Hughes and Beth Lewis Harkin in OMEGA - Journal of Death and Dying
