# Supplementary material for: The Impact of Continuing Bonds Between Pet Owners and Their Pets Following the Death of Their Pet: A Systematic Narrative Synthesis
Source: Omega (Westport). 2022 Sep 7;90(4):1666–84. doi: 10.1177/00302228221125955 (PMC11776356; doi:10.1177/00302228221125955)
Supplement: Supplemental Material - The Impact of Continuing Bonds Between Pet Owners and Their Pets Following the Death of Their Pet: A Systematic Narrative Synthesis [file sj-pdf-1-ome-10.1177_00302228221125955.pdf]

Appendix 1 – Literature assessment framework (Hawker et al., 2002)

Author and title:

Date:

	Good	Fair	Poor	Very poor	Comments
1. Abstract and title					
2. Introduction and aims					
3. Method and data					
4. Sampling					
5. Data analysis					
6. Ethics and bias					
7. Findings/results					
8. Transferability/generalizability					
9. Implications					
Total					

Appendix 2 – Literature scoring system (Hawker et al., 2002)

1. Abstract and title: Did they provide a clear description of the study?	
Good	Structure abstract with full information and clear title
Fair	Adequate abstract with most of the information
Poor	Inadequate abstract
Very poor	No abstract
2. Introduction and aims: Was there a good background and clear statement of the aims of the research?	
Good	Full but concise background to discussion/study containing up-to-date literature review and highlighting gaps in knowledge<br>Clear statement of aim AND objectives including research question
Fair	Some background and literature review; research questions outlined
Poor	Some background but no aim/objectives/questions, OR aims/objectives but inadequate background
Very poor	No mention of aims/objectives; no background or literature review
3. Method and data: Is the method appropriate and clearly explained?	
Good	Method is appropriate and described clearly (e.g., questionnaires included)<br>Clear details of the data collection and recording
Fair	Method appropriate, description could be better<br>Data described
Poor	Questionable whether method is appropriate<br>Method described inadequately<br>Little description of data
Very poor	No mention of method AND/OR method inappropriate AND/OR no details of data
4. Sampling: Was the sampling strategy appropriate to address the aims?	
Good	Details (age/gender/race/context) of who was studied and how they were recruited<br>Why this group was targeted<br>The sample size was justified for the study<br>Response rates shown and explained
Fair	Sample size justified<br>Most information given but some missing
Poor	Sampling mentioned but few descriptive details
Very poor	No details of sample
5. Data analysis: Was the description of the data sufficiently rigorous?	
Good	Clear description of how the analysis was done<br>Qualitative studies: description of how themes derived/respondent validation or triangulation<br>Quantitative studies: reasons for tests selected hypothesis driven/numbers add up/statistical significance discussed
Fair	Quantitative: descriptive discussion of analysis<br>Quantitative
Poor	Minimal details about analysis
Very poor	No discussion of analysis
6. Ethics and bias: have ethical issues been addressed and what necessary ethical approval gained? Has the relationship between researchers and participants been adequately considered?	

Good	Ethics: where necessary, issues of confidentiality, sensitivity and consent were addressed<br>Bias: Researcher was reflexive and/or aware of own bias
Fair	Lip service was paid to above (i.e., these issues were acknowledged)
Poor	Brief mention of issues
Very poor	No mention of issues
7. Results: Is there a clear statement of the findings?	
Good	Findings explicit, easy to understand, and in logical progression<br>Tables, if presents, are explained in text<br>Results relate directly to aims<br>Sufficient data are presented to support findings
Fair	Findings mentioned but more explanation could be given<br>Data presented relate directly to results
Poor	Findings presented haphazardly, not explained, and do not progress logically from results
Very poor	Findings not mentioned or do not relate to aims
8. Transferability or generalizability: Are the findings of this study transferable (generalizable) to a wider audience?	
Good	Context and setting of the study is described sufficiently to allow comparison with other contexts and settings, plus high score in Question 4 (sampling)
Fair	Some context and setting described but more needed to replicate or compare the study with others, PLUS fair score or higher in Question 4
	Minimal description of context/setting
Poor	No description of context/setting
Very poor	
9. Implications and usefulness: how important are these findings to policy and practice?	
Good	Contributes something new and/or different in terms of understanding/insight or perspective<br>Suggests ideas for further research<br>Suggests implications for policy and/or practice
Fair	Two of the above
Poor	Only one of the above
Very poor	None of the above

Appendix 3 – Characteristics of included studies table

Study details	Aims and objectives	Study design	Participant characteristics	Country	Setting	Theme(s) of included papers	Included/ excluded (Assessment Framework score)
Bereavement following death of a pet (Archer & Winchester, 1994)	To investigate the occurrence of grief following the death of a pet	Questionnaire	• (n=88) Experienced the death of a pet dog or cat within a year of the study	UK	Veterinary surgeries, local hairdressers, and social services	• Intensity of grief<br>• Support mechanisms and means of coping	Included<br>Hawker et al. assessment framework score<br>(20/36)
Pet Bonding and Pet Bereavement among Adolescents (B. H. Brown et al., 1996)	Descriptive data about pet ownership among adolescents and the degree on bonding by various animal types	Questionnaire	• Adolescents (12-17 years)<br>• Females (n=28); males (n=27)<br>• Mostly middle class<br>• All but 4 were white<br>• Had experienced the death of a pet within the year previous to the study	US	Boy Scouts, high schools, and referrals from friends and family	• Intensity of grief<br>• Support mechanisms and means of coping	Included<br>Hawker et al. assessment framework score<br>(31/36)
"My pet has passed":	To examine attachme	Questionnaires	• Undergraduate students	US	University	• Intensity of grief	Included

Relations of adult attachment styles and current feelings of grief and trauma after the event "(O. K. Brown & Symons, 2016)	nt relationships and emotional distress after the death of a pet		(Psychology, Kinesiology, nutrition, and a variety of arts and science disciplines<br>• 17-26 years old<br>• 82% females<br>• 93% Caucasian, 3% African Canadian, 3% Asian Canadian, 1% First Nations<br>• All had experienced a pet death within the last 5 years				Hawker et al. assessment framework score<br>(28/36)
Self-Compassion, Social Constraints, and Psychosocial Outcomes in a Pet Bereavement Sample (Bussolari	• To investigate social contexts and individual differences in experiences of pet bereavement<br>• The	Online survey/ questionnaire	• Cat and dog owners (at least 18 years old) whose pet had died 6 months before the study began<br>• 86.3%	US	Bereavement support groups	• Intensity of grief<br>• Support mechanisms and means of coping	Included<br>Hawker et al. assessment framework score<br>(28/36)

et al., 2018)	relationship between self-compassion and the frequency of continuing bonds efforts<br>• The degree to which self-compassion moderates the relationship between grief intensity and psychosocial outcomes<br>• The degree to which self-compassion moderates the relationship between socially constrained grief experience and psychosocial outcomes		female<br>• 34.1% had attended graduate school and an additional 52.2% had completed an undergraduate college degree<br>• Most participants (62.2%) were married or partnered and most did not have children (60.1%)<br>• 88.9% of participants self-identifying as White and Non-Hispanic.<br>• Just over half (56.7%) of the respondents endorsed a spiritual or religious				

			practice, with 30.1% identifying as Christian, 15.5% identifying as Catholic, 5.2% identifying as spiritual but not religious, 3.5% identifying as Jewish, and the remaining 7% identifying as Buddhist, New Age, Unitarian Universalist, or another religious/spiritual identity.				
Social Work with a Pet Loss Support Group in a University Veterinary Hospital (Dunn et al., 2005)	The development of a bereavement support group for pet owners in a university veterinary	Participation/ observation	• Group size of 8-12 people<br>• Mixture of ages, genders, religions, and races	US	Veterinary hospital	• Support mechanisms and means of coping	Included<br><br>Hawker et al. assessment framework score<br><br>(20/36)

	hospital.						
Grief Following Pet and Human Loss: Closeness is Key (Eckerd et al., 2016)	To compare grief severity between pet and human death and compare the predictors of grief severity for each	Questionnaires	• n=357 undergraduate students<br>• Aged 18-25<br>• Mainly female (71.7%)	US	University	• Intensity of grief	Included<br><br>Hawker et al. assessment framework score<br><br>(29/36)
Role of attachment in response to pet loss (Field et al., 2009)	To explore the impact of attachment on grief severity follow the death of a pet	Survey/ questionnaire	• 71 participants, 18+ years, whose cat or dog had died/separation within the past year<br>• Females (n=61); males (n=10)<br>• Married (n=48); Divorced (n=5); Widowed (n=2); single (n=14); other (n=2)<br>• Living alone (n=32); not living alone	US	Pet loss counsellors/ establishments	• Intensity of grief<br>• Support mechanisms and means of coping	Included<br><br>Hawker et al. assessment framework score<br><br>(31/36)

			(n=39)<br>• High school education (n=15); college education (n=34); graduate school (n=22)<br>• Buddhist (n=3); (Catholic n=21); Jewish (n=7); Protestant (n=15); non-denominational (n=25)				
Investigating the Emotion Regulation Strategies Implemented by Adults Grieving the Death of a Pet in Australia and the UK (Green et al., 2018)	• To investigate whether the ambiguous death of a pet is associated with heightened grief compared to an expected death<br>• To examine whether adaptive emotion regulation	Online survey	• n=134 individuals aged 18+ whose pet had died within 2-24 months at the time of recruitment<br>• Caucasian (83.6%)<br>• Female (84.3%)<br>• Majority were not grieving their first pet	Australia and UK	Online:<br>a. social media<br>b. online pet loss forums<br>c. advertisements to pet-related services, such as vets<br>d. An undergrad	• Intensity of grief<br>• Support mechanisms and means of coping	Included<br><br>Hawker et al. assessment framework score<br><br>(35/36)

	strategies are associated with less pet grief reactions than maladaptive strategies		loss (72.4%)		uate psychology unit at an Australian university		
Continuing Bonds and Psychosocial Functioning in a Recently Bereaved Pet Loss Sample (Habarth et al., 2017)	To understand the comforting factor of continuing bonds	Online survey	• n=4,336 who had lost a pet within the last year	US	• Pet bereavement support groups and associated websites	• Intensity of grief<br>• Support mechanisms and means of coping<br>• Personal growth	Included<br><br>Hawker et al. assessment framework score<br><br>(33/36)
Psychological sequalae of pet loss following Hurricane Katrina (Hunt et al., 2008)	To assess the psychological impact of pet loss on the survivors of Hurricane Katrina	Online questionnaires	• 65 participants<br>• White (n=62); African American (n=1); Asian American/Pacific Islander (n=1)<br>• Females (n=62); Males (n=3)<br>• Mean age – 37 years<br>• College-educate	US	Louisiana, Mississippi, and Alabama where Hurricane Katrina hit	• Intensity of grief	Included<br><br>Hawker et al. assessment framework score<br><br>(30/36)

			d (n=47).<br>• Income levels – between $15,000 – $45,000 /year (n=36); $45,000 /year or more (n=16); $15,000 /year or less (n=12).<br>• Single (n=21); in a relationship (n=14); married (n=21); divorced (n=8); widowed (n=1)<br>• Cat owners (n=27); dog owners (n=23); Owned both pets (n=14); parrot owner (n=1)				
Development of the Pet Bereavement Questionnaire (Hunt & Padilla,	To validate a bereavement scale, specific to people who have experienc	Devised by the authors and tested online	• n=142<br>• Mostly Caucasians (89%)<br>• Mostly female (89%)<br>• Mean age=42	US	• Websites devoted to pet bereavement<br>• Website for more	• Intensity of grief<br>• Support mechanisms and means of coping	Included<br><br>Hawker et al. assessment framewor

2006)	ed pet loss		years (SD= 10.7) with ages ranging from 18 to 63 years.<br>• unemployed (20%), employed (73%), students (7%).<br>• Of those who indicated their marital status: single (30%), married (59%), divorced (11%).<br>• Most respondents had lost a dog (67%) or a cat (29%), with other animal types being a small minority (4%): birds, rabbits and guinea pigs		general pet interest		k score (31/36)
And Then the Dog	To address	Autobiographical	• n=1 – an only	US	N/A	• Support mechani	Included

Died. (Kaufman & Kaufman, 2006)	childhood pet bereavement in the context of multiple prior losses	case analysis	child born to older parents			sms and means of coping<br>• Personal growth	Hawker et al. assessment framework score<br><br>(28/36)
Attachment, Social Support, and Responses Following the Death of a Companion Animal (King & Werner, 2011)	To test hypotheses concerning attachment, social support, and<br><br>grief responses to the loss of animal companionship	Questionnaire	• n=429<br>• Female (87.9%); male (12.1%) men<br>• Mean age of 41.55 years (SD = 11.42)<br>• The majority of participants were White (n = 388; 90.4%) and college educated (n = 386; 90.0%)<br>• Most (366; 85.3%) were from diverse regions within the US, but 35 (8.2%) were from Canada,	US, Canada, UK, Australia, New Zealand	• Pet loss support websites<br>• General pet-related internet sites<br>• Internet classified adverts<br>• Printed recruitment announcements posted at veterinary clinics and hospitals, companion animal adoption sites, and a university campus	• Intensity of grief<br>• Support mechanisms and means of coping	Included<br><br>Hawker et al. assessment framework score<br><br>(31/36)

			12 (2.8%) were from the UK, 8 (1.9%) were from Australia, and 8 (1.9%) were from New Zealand.				
Situational factors related to loneliness and loss over time among older pet owners (Krause-Parello & Gulick, 2013)	To examine the interrelations of situational factors, coping, and pet attachment that affect loneliness in 191 older adults who experienced loss over time.	Survey/ questionnaire	• Females (n=159); males (n=32)<br>• White (n=184); Black/African American (n=3); Hispanic/Latino (n=2); Native American/Alaskan Native (n=1); Other (n=1)<br>• No formal school (n=1); some grammar school (n=4); completed grammar school (n=6); some high school	US	Community	• Intensity of grief<br>• Support mechanisms and means of coping	Included<br><br>Hawker et al. assessment framework score<br><br>(29/36)

			(n=30); completed high school (n=64); some college (n=45); completed college (n=41)<br>• Widowed (n=75); married/partnered (n=56); divorced (n=45); never married (n=13); separated (n=2)<br>• Lives alone (n=117); lives with spouse/significant other (n=55); lives with family/relative (n=15); lives with friend (n=3); lives with parent (n=1)<br>• Lives in 55 and over				

			community residence (n=151); lives in private community residence (n=40)<br>• Cat owner (n=97); dog owner (n=94)				
"They Burn Brightly, But Only for a Short Time": The Role of Social Workers in Companion Animal Grief and Loss (Laing & Maylea, 2018)	To explore whether there was a role for social work practitioners to support grieving animal companion guardians.	Online responses to an article about losing a companion animal	Sample size (n=218) mentioned – no details provided	Australia	Online community	• Intensity of grief<br>• Support mechanisms and means of coping	Included<br><br>Hawker et al. assessment framework score<br><br>(26/36)
Grief Severity: A Comparison Between Human and Companion Animal Death (Lavorgna & Hutton,	To build on the limited existing literature comparing the grief experience of human and	Questionnaire	• n=50<br>• Aged 22-66<br>• Females (82%); males (18%)	Australia	The Australian Centre of Grief and Bereavement and the Australian College of Applied Psychology via	• Intensity of grief<br>• Support mechanisms and means of coping	Included<br><br>Hawker et al. assessment framework score<br><br>(30/36)

2019)	companion loss				flyers, social media and word of mouth		
Religion and Pet Loss: afterlife beliefs, religious coping, prayer and their associations with sorrow (Lee, 2016)	To examine the relationship between religion and sorrow among owners of deceased pets	Online survey	• Participants were self-selected<br>• White (90.4%)<br>• Female (85.84%)<br>• Married /cohabiting (58.0%),<br>• Educated at the Bachelor's level or beyond (56.6%)<br>• Christian (61.6%)<br>• Mean age of 41.26 (SD=11.73) years.<br>• Deceased pets: dogs (63.9%), euthanised (65.3%) and died within one week of the survey (71.2%).	US	Pet bereavement website	• Intensity of grief<br>• Support mechanisms and means of coping<br>• Personal Growth	Included<br><br>Hawker et al. assessment framework score<br><br>(27/36)
Neuroticism and Religious Coping	To examine the influence	Online survey	• Females (n=417); males (n=90)	US	Online community	• Intensity of grief<br>• Support mechani	Included<br><br>Hawker et

Uniquely Predict Distress Severity among Bereaved Pet Owners (Lee & Surething, 2013)	of neuroticism and religious coping on pet bereavement distress		• White (n=460); Black (n=5); Asian (n=13); Hispanic (n=18) • Education – less than grade 12 (n=6); high school (n=55); college/ technical school (n=141); university (n=139); postgraduate (n=168) • Divorced /separated (n=167); married/ living with partner (n=308); widowed (n=4); single (never married) (n=129) • Christians (n=280); atheists (n=97); Jews (n=44); Buddhists (n=14); Muslims			sms and means of coping	al. assessment framework score (32/36)

			(n=10); Hindus (n=3); others (n=2)<br>• Pet type – dog (n=309); cat (n=188); bird (n=3); other pets – no (n=154); yes (n=355)<br>• Death type – non-euthanised (n=172); euthanised (n=334)				
Complicated Grief and Post-Traumatic Stress Disorder in Humans' Response to the Death of Pets/Animals (Luiz Adrian et al., 2009)	To determine the percentage of people reporting significant sympoms of complicated grief and/or PTSD following the death of a pet	Self-report survey	• n=106<br>• Female (n=81); male (n=25)<br>• Ethnicity – Asian/Asian-American (n=30); European/white/Caucasian (n=46); Hispanic/Latino (n=7); Native Hawaiian (n=15);	US	Veterinary clinics	• Intensity of grief<br>• Support mechanisms and means of coping	Included<br><br>Hawker et al. assessment framework score<br><br>(31/36)

			Pacific Islander (n=2); Other (n=4)<br>• Lived on the big island of Hawaii for most of life: No (n=48); yes (n=58)<br>• Marital status: single, never married (11); married/ living with partner (n=80); divorced /separated (n=10); widowed (n=5)<br>• Annual household income: Less than $20,000 (n=6); $20,000-$39,999 (n=18); $40,000-$74,999 (n=40); $75,000 and over (n=36)<br>• Level of educatio				

			n completed: Some high school (n=2); high school diploma (n=21); some college (n=35); college degree (n=34); some postgraduate (n=3); postgraduate (n=10); other (n=1)				
Grief Resulting from Euthanasia and Natural Death of Companion Animals (McCutcheon & Fleming, 2001)	To investigate the influence of a number of variables on how someone adjusts to companion-animal death	Questionnaire	• n=103 • Aged 18+: 18-35 (n=27; 26.2%); 36-59 (n=56; 54.4%); 60 and over (n=20; 19.4%) • Female (n=74; 71.8%); male (n=29; 28.2%)	Canada	Veterinary clinic/holistic centre in Toronto	• Intensity of grief • Support mechanisms and means of coping	Included<br><br>Hawker et al. assessment framework score<br><br>(26/36)
Managing Pet Owners' Guilt and Grief in	To understand how medical profession	Ethnography – observations and interviews	Very few details – • 81 interviews with	US	Large veterinary teaching hospital in New York	• Support mechanisms and means of coping	Included<br><br>Hawker et al.

Veterinary Euthanasia Encounters (Morris, 2012)	als manage the emotions of their clients		54 vets and over 600 hours of observation<br>• 70% of interviewees were female, experiences ranged from novices to nearly 40 years				assessment framework score<br>(22/36)
Continuing Bonds and Psychosocial Adjustment in Pet Loss (Packman et al., 2011)	To investigate continuing bonds expressions among bereaved pet owners and the relationship between CB expressions and psychosocial adjustment.	Questionnaires and interview	• n=33<br>• Aged 25-79 (average age of 45.57)<br>• Females (n=27; 81.8%); males (n=6; 18.2%)<br>• Marital status: single (n=13; 39.4%); married/partnered (n=15; 45.5%); divorced (n=5; 15.2%)<br>• Highest level of education: high school (n=2; 6.1%); college (n=13;	US	Veterinary clinics, family centres, and at a pet loss support group.	• Intensity of grief<br>• Personal growth	Included<br>Hawker et al. assessment framework score<br>(32/36)

			39.4%); graduat e school (n=18; 54.5%)<br>• Racial/et hnic backgro und: Latino (n=2; 6.1%); Caucasia n (n=27; 81.8%); Asian/Pa cific Islander (n=1; 3%); Wiccan (n=1; 3%); other (n=2; 6.1%)<br>• Annual househo ld income: Less than $25,000 (n=5; 15.2%); $25,000- $49,000 (n=6; 18.2%); $50,000- $74,999 (n=5; 15.2%); $75,000- $100,00 0 (n=7; 21.2%); more than $100,00 0 (n=10;				

			30.3%)				
Therapeutic implications of continuing bonds expressions following the death of a pet (Packman et al., 2012)	To demonstrate how the "continuing bonds" concept applies to the human-pet relationship and describe the unique, ongoing relation-ships and bonds formed by bereaved pet owners following pet loss.	Interview	• n=33, at least 18 years of age and had lost a pet within 12 months from the date of data collection.<br>• Females (n=27); males (n=6)<br>• Latino (n=2); Caucasian (n=27); Asian/Pacific Islander (n=1); Wiccan (n=1); other (n=2)<br>• Single (n=13); married/partnered (n=15); divorced (n=5)<br>• Highest levels of education – high school (n=2); college (n=13); graduate school (n=18)	US	Various locations such as veterinary clinics, family centres, practicum and internship sites, and at a pet loss support group.	• Intensity of grief<br>• Support mechanisms and means of coping	Included<br><br>Hawker et al. assessment framework score<br><br>(32/36)

			• Household total yearly income – Less than $25,000 (n=5); $25,000-$49,000 (n=6); $50,000-$74,999 (n=5); $75,000-$100,000 (n=7); over $100,000 (n=10) • Type of pet – dog (n=19); cat (n=14) • Cause of death (could be multiple) – Natural/anticipated (n=9); unexpected (5); major disease (n=23); other (n=7)				
Online survey as empathetic bridging for the loss of disenfranchised grief	Investigated grief reactions of bereaved individuals following the death	Survey (option of online or hard copy)	• 235 participants selected from a total of 3,363 respondents	US and French Canadian	Online and pet loss support groups	• Intensity of grief • Support mechanisms and means of coping • Personal	Included<br><br>Hawker et al. assessment framewor

of pet loss (Packman et al., 2014)	of a pet		• Females (n=203); males (n=32) • Single (n=81); married/ partnered (n=129); divorced (n=20); separated (n=2); widowed (n=3) • Children – yes (n=81); no (n=154) • Relationship to deceased – Best friend (n=92); parental (n=79); partner/ significant other (n=36); other (n=28) • Education – less than high school (n=3); high school (n=35); vocational/trade school (n=16); college (n=110); graduat			growth	k score (31/36)

			e school (n=71)<br>• Income – Less than \$25,000 (n=21); \$25,000-\$49,999 (n=61); \$50,000-\$74,999 (n=49); \$75,000-\$100,000 (n=41); more than \$100,000 (n=63)<br>• Cause of death – Natural (n=34); unexpected (n=63); major disease (n=76); other (n=62)<br>• Euthanasia -Yes (n=155); no (n=80)				
Posttraumatic growth following the death of a pet (Packman et al., 2017)	To examine posttraumatic growth (PTG) experienced by bereaved pet owners following	Survey (option of online or hard copy)	• Females (n=251); males (n=57)<br>• Marital status – Single (n=83); married/partnered (n=176); divorced (n=38);	US	Online and pet loss support groups	• Support mechanisms and means of coping<br>• Personal growth	Included<br><br>Hawker et al. assessment framework score<br><br>(33/36)

	the death of their pet.		separated (n=8); widowed (n=3)<br>• African American (n=2); Latino (n=15); Native American (n=4); Caucasian (n=268); Asian Pacific Islander (n=10); Multi-ethnic (n=2); other (n=7)<br>• Age of owners – mean=42 years<br>• Age of pet – mean=11 years<br>• Education – less than high school (n=3); high school (n=30); vocational/trade school (n=15); college (n=65); graduate school (n=91)				

			• Income – Less than $25,000 (n=22); $25,000-$49,999 (n=66); $50,000-$74,999 (n=69); $75,000-$100,000 (n=72); more than $100,000 (n=0)<br>• Relationship to deceased – Best friend (n=225); parental (n=95); partner/ significant other (n=75); other (n=88)<br>• Most important role – Best friend (n=138); parental (n=102); partner/ significant other (n=34); other (n=34)<br>• Cause of death – Natural (n=54); unexpec				

			ted (n=71); major disease (n=121); other (n=62)<br>• Euthanasia – Yes (n=213); no (n=95)<br>• Children – yes (n=120); no (n=188)<br>• Spiritual practice – yes (n=212); no (n=197)				
Pet grief: when is non-human life grievable? (Redmalm, 2015)	An exploration of how pet owners grieve their pets and view their pet's transience	Interviews	• n=18<br>• Females (n=11); males (n=7)<br>• Age – 20-70 years old; half were under 40<br>• Relationship status – Single (n=6); had children (n=9)<br>• Location living in – Rural area (n=7); non-rural area	Sweden	Interviewees' homes	• Intensity of grief	Included<br><br>Hawker et al. assessment framework score<br><br>(25/36)

			(n=11)<br>• Education – Qualified employment (n=12)<br>• Place of birth – Sweden (n=15); outside Sweden (n=3)<br>• Animal(s) owned – Dog (n=11); cat (n=7); bird (n=2); fish (n=1); rat (n=1); snakes and lizards (n=1)				
Exploring the Grief Experience Among Callers to a Pet Loss Support Hotline (Rémillard et al., 2017)	To explore the grief experience among callers<br><br>to the Ontario Veterinary College Pet Loss Support Hotline (PLSH)	Retrospective content analysis of call notes	• n=65<br>• No other information provided /available	Canada	Retrospective analysis – one of the authors retrieved the call data/notes	• Intensity of grief<br>• Support mechanisms and means of coping<br>• Personal growth	Included<br><br>Hawker et al. assessment framework score<br><br>(26/36)
In praise of dead pets: an	To assess the value of eulogy	Interpretive ontology informed	• 19 eulogies – dogs (n=9);	UK (n=7), New Zealand	Correspondence	• Intensity of grief<br>• Support mechani	Included

investigation into the content and function of human-style pet eulogies (Rennard et al., 2019)	writing as a therapeutic memorial device for bereaved pet owners.	an inductive thematic analysis of eulogies	cats (n=8); rabbits (n=1); pony (n=1)	(n=5), US (n=3); Australia (n=3); South Africa (n=1)		sms and means of coping	Hawker et al. assessment framework score (34/36)
Veterinarians' views on pet loss: evidence from Romania (Rujoiu & Rujoiu, 2015)	Unclear but mentions 2 questions: 1. What happens when the ''clients'' are actually the veterinarians? 2. What do Romanian veterinarians think and how do they relate to the euthanasia of their animal companion and to pet loss counsell	In-depth interviews – face-to-face (n=14); telephone (n=1); in writing (n=5)	• 20 participants • Females (n=8); males (n=12) • Aged 26-56 years old	Romania	• Private clinic (n=9) • Veterinary state facility (n=1)	• Intensity of grief • Support mechanisms and means of coping	Included Hawker et al. assessment framework score (24/36)

	ing?						
Pet loss and continuing bonds in children and adolescents (Schmidt et al., 2020)	An exploration of the way children use continuing bonds to cope following the death of a pet	Children – questionnaires and interview<br><br>Parents – demographic questionnaire	• 32 children (aged 5-18 years) and their parents<br>• Females (n=20); males (n=12)<br>• Race/ethnicity – White/Caucasian (59.4%); Latino/Hispanic (21.9%); 9.4% Native American (9.4%); Asian/Pacific Islander (9.4%)<br>• Average number of days since the death of the pet was 40.91 (SD 1⁄4 38.92, Range 1⁄4 3–135)<br>• On average, pets were 7.91 years old at the time of death	US	Pet loss support organisations, veterinary clinics, schools, and internet listing<br><br>sites (e.g. Craigslist, Listserve, and Pet Loss Websites)	• Intensity of grief<br>• Support mechanisms and means of coping	Included<br><br>Hawker et al. assessment framework score<br><br>(30/36)

			(SD 1⁄4 5.08, Range 1⁄4 0.42–15.5).<br>• Number of pets each child had lost over their lifetime ranged from 1 to 21 (M 1⁄4 2.94, SD 1⁄4 4.89)				
Pet Loss and Representations of Death, Attachment, Depression, and Euthanasia (Testoni et al., 2017)	• To describe the relationship between ontological representation of death and attachment using a structural model in which the representation of death as annihilation (the negation of any after-life) and the attachment of	Questionnaire	• 159 participants<br>• Females (n=111); males (n=48)<br>• Mean age – 45 years (SD = 14.7), with ages ranging from 18 to 79<br>• Education – university degree (39.6%); high school degree (49.7%); middle school education (7.5%); element	Italy	Word of mouth, veterinary clinics, pet stores, and animal welfare associations	• Intensity of grief<br>• Support mechanisms and means of coping	Included<br><br>Hawker et al. assessment framework score<br><br>(32/36)

	the owner to the pet were the independent variables.<br>• To examine the management of euthanasia and the relationship between the pet owner and the veterinarian.		ary school education (2.5%); uneducated (0.6%)<br>• Employed (72%)<br>• Married or cohabitating (52%); single (34%); separated/divorced (9%); widowed (5%)<br>• Had children (46%)<br>• Housing conditions – Lived with at least one other adult but no children (49%); lived alone (28%); lived with adults and children (22%); lived only with children (1%)<br>• Beliefs –				

			Belief in God (59%); belief in an afterlife (66%). Of these Respondents – believed an afterlife was only for humans (13%); believed it was for both people and animals<br>• Death of animal – dog (69%); cat (25%); other animals, including horses, birds, and ferrets (6%)<br>• The pet's death occurred two years before the interview (65%)<br>• The mean age of the pet				

			at death was 11 years				
Grief and Bereavement of Israeli Dog Owners: Exploring Short-Term Phases Pre- and Post-Euthanization (Tzivian et al., 2014)	To explore the emotional and cognitive processes of bereaved dog owners	Semi-structured interviews	• n=29<br>• Female (n=24; 82.8%); male (n=5; 17.2%)<br>• Family status: single (n=6; 20.7%); married (n=17; 58.6%); divorced (n=3: 10.3%); widowed (n=3: 10.3%)<br>• Education: 8-12 years (n=9; 31%); 12+ years (n=20; 69%)<br>• Kind of work: physical (n=1; 34.4%); office (n=12; 41.4%); housewife (n=2; 6.9%); nonworking (n=3; 10.3%); artists (n=11; 37.9%)<br>• Financial	Israel	Participants were recruited from central and southern Israel	• Intensity of grief<br>• Support mechanisms and means of coping	Included<br><br>Hawker et al. assessment framework score<br><br>(32/36)

			status: less than average (n=3; 10.3%); average (n=14; 48.3%); more than average (n=12; 41.4%)				
Association Between Stress and Quality of Life: Differences Between Owners Keeping a Living Dog or Losing a Dog by Euthanasia (Tzivian et al., 2015)	To compare the levels of stress and to explore the correlates of QOL of healthy adults who currently own or who have just lost their dog.	Questionnaire	• n=213<br>• All female, healthy, bereaved dog owners whose dogs were euthanised	Israel	Participants were recruited from central and southern Israel	• Intensity of grief<br>• Support mechanisms and means of coping	Included<br><br>Hawker et al. assessment framework score<br><br>(34/36)
Beyond Recovery: Understanding the Post-bereavement Growth from Companion Animal Loss (Wong et al., 2017)	To explore animal companion experiences of animal loss in Chinese society	In-depth interviews	• n=31<br>• Female (77%); male (23%)<br>• Deceased animal: dog (n=19); cat (n=8); rabbit (n=2); bird (n=1)	Hong Kong	Convenience and snowball sampling through animal welfare agencies, veterinary clinics, funeral parlours for animals,<br><br>and	• Intensity of grief<br>• Support mechanisms and means of coping<br>• Personal growth	Included<br><br>Hawker et al. assessment framework score<br><br>(32/36)

					Animal Power (a non-governmental agency in Hong Kong that specializes in providing individual and group bereavement counselling to people bereaved by animal loss)		
Grieving Pet Death: Normative, Gender and Attachment Issues (Wrobel & Dye, 2003)	To investigate grief over the loss of a pet to clarify the usual course of symptoms experienced, gender differences in the experience, and the role of attachment to the	Surveys	• n=174<br>• Female (n=112); male (n=62)<br>• Aged 18-85, with a mean age of 35 years.<br>• The majority of the sample was Caucasian (90.2%), with 4.6% African American, 1.1% America	US	A college campus, a local business setting, and referrals from various local veterinarians	• Intensity of grief<br>• Support mechanisms and means of coping	Included<br><br>Hawker et al. assessment framework score<br><br>(25/36)

	pet.		n Indian, and 2.9% Asian<br>• Marital status: Married (36.8%); single (55.7%); separated (1.7%); divorced (5.2%); widowed (6%)<br>• Living arrangements: lived alone 15.5% lived alone<br>• Children: only a minority (32.8%) had children<br>• Education: less than high school (2%); high school graduates (15.5%); some college education (54.6%); college degree (14.9%); advanced degree				

			(10.3%)<br>• Work: outside the home (82.2%); not working (17.8%)<br>• Animal death: dog (69.5%); cat (30.5%)				
An attachment perspective on human-pet relationships: Conceptualisation and assessment of pet attachment orientations (Zilcha-Mano et al., 2011)	To examine human-pet relationships<br><br>Study 5 – To examine whether and how individual differences in pet attachment orientations contribute to the process of grieving following the loss of a pet.	Questionnaires	• n=137<br>• Female (n=850; male (n=52)<br>• Aged 17-71 (M = 31, SD = 9.02)<br>• The duration of the relationship with the lost pet ranged from 2.4 to 19.5 years (M=8.6, SD=5.17).<br>• Participants lost their pets for various reasons: sickness (34.75%), traffic accidents (14.89%),	Israel	Participants were recruited in parks, animal food and equipment stores, universities, and malls in the central area of Israel.	• Intensity of grief	Included<br><br>Hawker et al. assessment framework score<br><br>(29/36)

			euthanasia (29.79%), or old age (10.64%). Most of them had lost dogs (64.54%) or cats (26.95%)				
